# Developing immune-regulatory materials using immobilized monosaccharides with immune-instructive properties

**DOI:** 10.1016/j.mtbio.2020.100080

**Published:** 2020-09-30

**Authors:** M.A. Alobaid, S.-J. Richards, M.R. Alexander, M.I. Gibson, A.M. Ghaemmaghami

**Affiliations:** a Immunology & Immuno-Bioengineering, School of Life Sciences, Faculty of Medicine and Health Sciences, University of Nottingham, Nottingham, NG7 2RD, United Kingdom; b Department of Chemistry, University of Warwick, Coventry, CV4 7AL, United Kingdom; c Warwick Medical School, University of Warwick, Coventry, CV4 7AL, United Kingdom; d School of Pharmacy, University of Nottingham, Nottingham, NG7 2RD, United Kingdom; e Terasaki Institute for Biomedical Innovation, Los Angeles, CA, 90024, USA

**Keywords:** Dendritic cells, T cells, Carbohydrates, Immune-instructive materials, Immune modulation, Fucose, Galactose, Mannose, Polymers, CLR, C-type lectin receptor, DCs, Dendritic cells, DC-SIGN, Dendritic cell-specific intercellular adhesion molecule 3-grabbing nonintegrin, IDO, Indoleamine 2,3-dioxygenase, MR, Mannose receptor, MFI, Median fluorescence intensity, PRR, Pattern recognition receptor, LPS, Lipopolysaccharide, FBS, Fetal bovine serum, MT, 1-methyl-DL-tryptophan, (Gal1), 100% 1-amino-1-deoxy-β-d-galactose, (Gal2), 100% 2-amino-2-deoxy-β-d-galactose, (Gal2–Man1), 90% 2-amino-2-deoxy-β-d-galactose + 10% 1-amino-1-deoxy-β-d-mannose, (Gal2–Man2), 2-amino-2-deoxy-β-d-galactose + 10% 2-amino-2-deoxy-β-d-mannose, (Man1–Man2), 40% 1-amino-1-deoxy-β-d-mannose + 60% 2-amino-2-deoxy-β-d-mannose, (Gal1–Gal2), 50% 1-amino-1-deoxy-β-d-galactose + 50% 2-amino-2-deoxy-β-d-galactose

## Abstract

New strategies for immune modulation have shown real promise in regenerative medicine as well as the fight against autoimmune diseases, allergies, and cancer. Dendritic cells (DCs) are gatekeepers of the immune system and their ability in shaping the adaptive immune responses makes DCs ideal targets for immune modulation. Carbohydrates are abundant in different biological systems and are known to modulate DC phenotype and function. However, how simple monosaccharides instruct DC function is less well understood. In this study, we used a combinatorial array of immobilized monosaccharides to investigate how they modulate DC phenotype and function and crucially the impact of such changes on downstream adaptive immune responses. Our data show that a selection of monosaccharides significantly suppress lipopolysaccharide-induced DC activation as evidenced by a reduction in CD40 expression, IL-12 production, and indoleamine 2,3-dioxygenase activity, while inducing a significant increase in IL-10 production. These changes are indicative of the induction of an anti-inflammatory or regulatory phenotype in DCs, which was further confirmed in DC–T cell co-cultures where DCs cultured on the ‘regulatory’ monosaccharide-coated surfaces were shown to induce naïve T cell polarization toward regulatory phenotype. Our data also highlighted a selection of monosaccharides that are able to promote mixed Treg and Th17 cell differentiation, a T cell phenotype expected to be highly immune suppressive. These data show the potential immunomodulatory effects of immobilized monosaccharides in priming DCs and skewing T cell differentiation toward an immune-regulatory phenotype. The ability to fine-tune immune responses using these simple carbohydrate combinations (e.g. as coatings for existing materials) can be utilized as novel tools for immune modulation with potential applications in regenerative medicine, implantable medical devices, and wound healing where reduction of inflammatory responses and maintaining immune homeostasis are desirable.

## Graphical abstract

**Figure:**
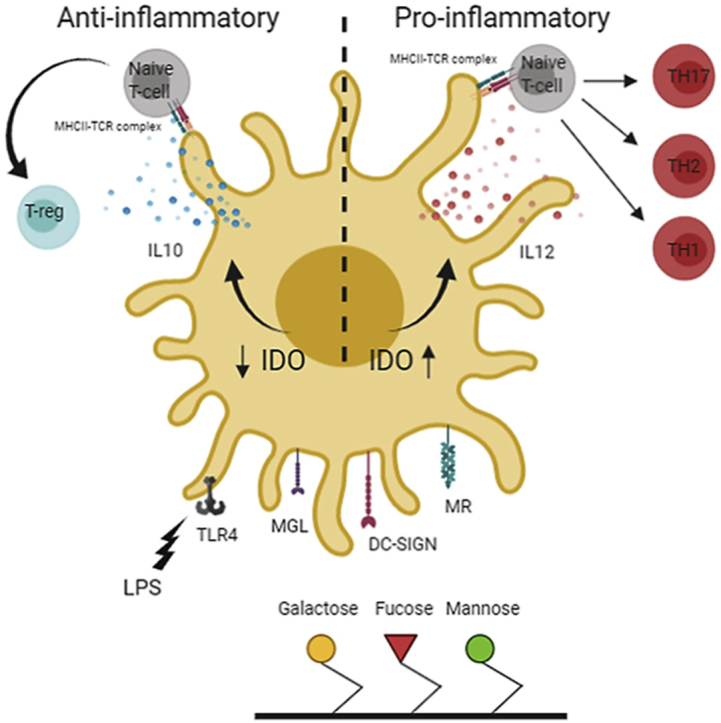

## 1 Introduction

The immune system plays a central role in response to foreign materials we are exposed to, from pathogens to biomaterials used for therapeutic purposes [1]. The ability to suppress adverse immune responses and promote beneficiary regulatory and pro-healing immune responses will improve the clinical outcome for implanted materials whether used as scaffolds for regenerative medicine applications or in medical devices [2]. Dendritic cells (DCs) are the immune system's sentinels that sense their surrounding environment using a host of different pattern recognition receptors (PRRs) [3]. Upon activation, DCs migrate to draining lymph nodes where they prime naïve T cells and instruct their differentiation toward different functional phenotypes (e.g. Th1, Th2, Treg, or Th17), a process that is heavily influenced by DCs' surface phenotype, cytokine profile, and expression of other immune-regulatory molecules [[4], [5], [6]]. DCs constantly sample their environment using PRRs, which enable them to transfer information about the nature of antigens and foreign materials they have encountered in peripheral tissues to T cells in lymph nodes [7]. Carbohydrates are an integral part of almost all proteins and play key roles in biological processes including self and non-self recognition pathways and their associated signaling pathways. The wide range of functions carried out by carbohydrates is reflected in their structural diversity [8,9]. The C-type lectin receptors (CLRs) are amongst many PRRs expressed by DCs and belong to a family of carbohydrate-binding proteins that recognize a broad repertoire of self and non-self ligands, with diverse functions in innate and adaptive immunity and maintaining homeostasis [10]. Different CLRs recognize different carbohydrate moieties, and interaction between a given CLR and its ligand could influence DC phenotype and cytokine profile as well as downstream events leading to T cell polarization [11,12]. Prime examples of these are the carbohydrate moieties decorating allergens and pathogen glycoproteins, which modulate the immune response depending on their CLR counterparts [[12], [13], [14], [15]] leading to inflammatory responses or immune evasion [[16], [17], [18]]. In parallel to their role in initiating immune responses, DCs also play a key role in maintaining tissue homeostasis and immune regulation. Immune regulatory enzymes, such as indoleamine 2,3-dioxygenase (IDO), have been implicated in these processes [19,20]. IDO is a rate-limiting enzyme that converts l-tryptophan into kynurenine, a metabolite with well-established immune-modulatory properties [13,21,22]. This is exemplified in tumors with high IDO activity leading to depletion of tryptophan and an increase in kynurenine levels that suppresses the immune activity that contributes to the survival of the tumor [23].

Different surface-modification strategies have been used to develop materials with higher immune compatibility or the ability to modulate immune responses [24]; however, despite their strong immune-modulatory properties, carbohydrates have not been extensively considered in this context. While extensive literature on the immune-modulatory properties of polysaccharides exist, the immune-modulatory potential of monosaccharides is not well studied. This is despite many advantages in using monosaccharides in immune modulation including the ability to synthesize, modify, and characterize highly pure preparations. In this study, we use a combinatorial library of polymer-tethered surface-immobilized monosaccharides (manno-, galacto-, and fuco-) and investigate their impact on DC phenotype, cytokine profile, and IDO activity, as well as the ability of carbohydrate primed DCs to instruct T cell differentiation toward distinct functional phenotypes.

## 2 Materials and methods

### 2.1 Generation of human DCs from peripheral blood monocytes

**Fig. S1:**
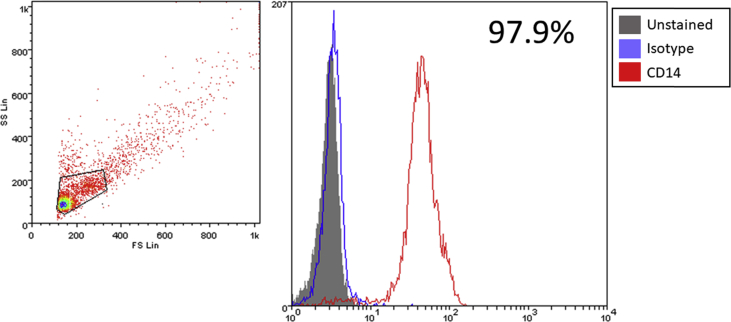
**Flow cytometry staining of isolated monocytes from buffy coat.** Data shows a purity of 97.9% CD14+ monocytes confirming the quality of the isolation process and the yield of target cells. Data show results from a representative experiment of >6 independent experiments.

**Fig. S2:**
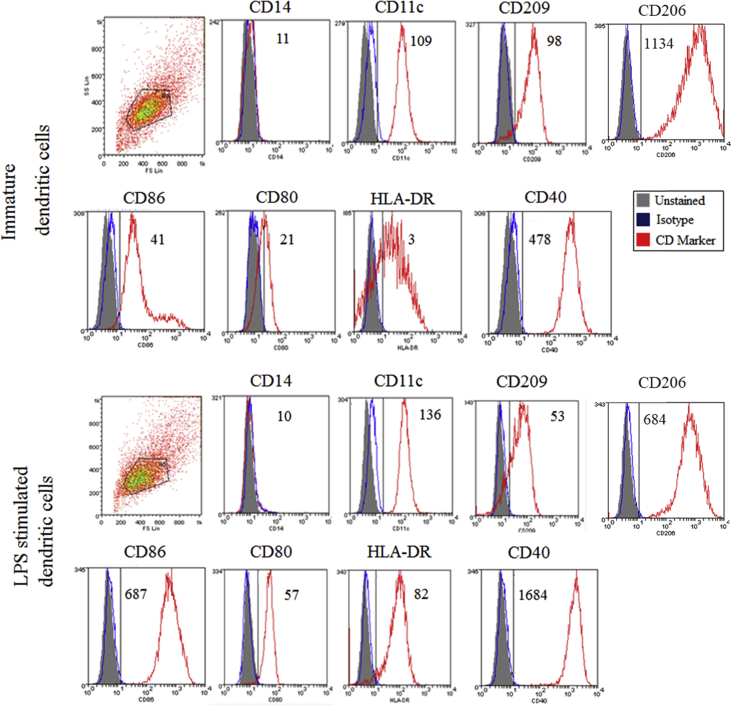
Flow cytometry phenotypic analysis of Immature and mature DCs where Immature dendritic cells generated show increased expression of CD209, CD206 CD11c, low expression of HLA-DR, CD80, CD86, CD40 and no expression of CD14 proving successful generation of immature dendritic cells on day 6. Following 10ng/ml E-coli LPS, stimulation expression of CD80, CD86, CD40 and HLA-DR increased while CD209, CD206 decreased and having maintained presence of CD11c with absence of CD14 show that dendritic cells were successfully matured. Data showing results in Median Fluorescence Intensity (MFI) from a representative experiment of >3 donors.

This was done as we have previously described [25]. Briefly, buffy coats were obtained from healthy donors following ethics committee approval and informed written consent (National Blood Service, UK). Separation of peripheral blood monocytes (PBMCs) was done by means of density gradient centrifugation using Histopaque (Sigma-Aldrich, UK). CD14^+^ monocytes were isolated using magnetic-assisted cell sorting (Miltenyi Biotec, UK). The purity of isolated monocytes was confirmed by flow cytometry and it was consistently >95% (Supplementary Fig. 1). Purified CD14 monocytes were supplemented with RPMI media containing 10% heat-inactivated fetal bovine serum (FBS), 100 mg/mL streptomycin, 100 U/mL penicillin, and 2 mM l-glutamine (all from Sigma-Aldrich). To generate immature DCs, monocytes were then seeded (1 × 10^6^ cells per well) in a 24-well tissue culture plate with the presence of 50 ng/mL GM-CSF and 250 U/mL IL-4 (both from Miltenyi Biotec) and incubated at 37 °C in a 5% CO_2_-humidified incubator for 6 days for differentiation, where fresh media was added on day 3. Immature DC phenotype and maturation were confirmed through flow cytometry (Supplementary Fig. 2).

### 2.2 Polymerization of *N-*hydroxyethyl acrylamide

*N*-Hydroxyethyl acrylamide (1 g, 8.69 mmol), pentafluorophenyl 2-(dodecylthiocarbonothioylthio)-2-methyl propionic acid (PFP-DMP) (0.1844 g, 0.35 mmol), and 4,4′-azobis (4-cyanovaleric acid) (ACVA) (0.0195 g, 0.069 mmol) were dissolved in 50:50 toluene:methanol (4 mL). Mesitylene (150 μL) was added as an internal reference. An aliquot was taken for NMR analysis in CDCl_3_. The solution was degassed under N_2_ for 30 min. The reaction was stirred at 70 °C for 90 min. An aliquot was taken for NMR analysis in MeOD to determine conversion. The reaction was rapidly cooled in liquid nitrogen and precipitated into diethyl ether. The polymer was reprecipitated into diethyl ether from methanol twice to yield a yellow polymer product that was dried under vacuum (97% conversion by NMR, M_n_ (theoretical) = 3,400 g/mol, Mn (SEC) = 4,100 g/mol, M_w_/M_n_ (SEC) = 1.14).

### 2.3 Synthesis of 1-amino-1-deoxy-mannose, -galactose, and -fucose

Galactose, mannose, and fucose (1 g) were stirred in 10 mL aqueous saturated ammonium carbonate for 2–5 days, ensuring the solution remained saturated. Reaction completion was determined by mass spectrometry. After completion of the reaction, the solution was freeze-dried. Excess ammonium carbonate was removed by addition of warm methanol. After complete evolution of CO_2,_ the methanol was removed *in vacuo.* ESI-MS: 1-amino-1-deoxy-mannose (C_6_H_13_NO_5_) M = 179.17 expected, [2M − H]^-^ observed 359.1 (C_12_H_25_N_2_O_10_), 1-amino-1-deoxy-galactose (C_6_H_13_NO_5_) M = 179.17 expected, [2M − H]^-^ observed 359.1 (C_12_H_25_N_2_O_10_), and 1-amino-1-deoxy-fucose (C_6_H_13_NO_4_) M = 163.17 expected, [M − H]^-^ observed 162.1.

### 2.4 End-group modification of PFP-poly(*N*-hydroxyethyl acrylamide) (PFP-pHEA) using amino monosaccharides

**Fig. 1:**
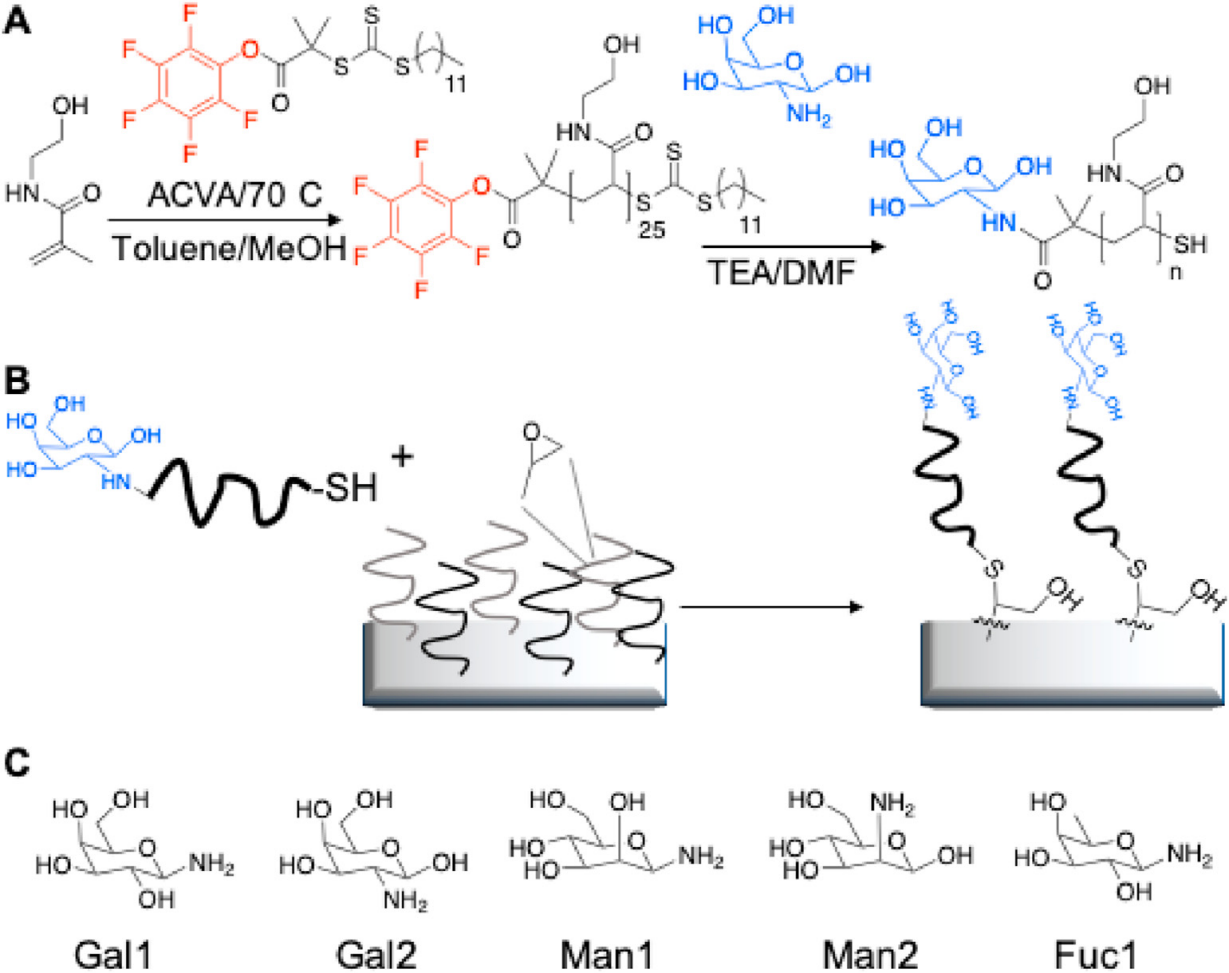
Synthetic route to carbohydrate-coated plates. (A) Polymerization of *N*-hydroxyethyl acrylamide *via* RAFT polymerization to a degree of 20 and subsequent carbohydrate functionalization of pHEA. (B) Immobilization of carbohydrate-terminated polymers onto epoxy-coated 96-well plates [33]. (C) Monosaccharides used in the study from left to right, 1-amino-1-deoxy galactose, 2-amino-2-deoxy galactose, 1-amino-1-deoxy mannose, 2-amino-2-deoxy mannose, and 1-amino-1-deoxy fucose.

In a typical reaction, PFP-pHEA (100 mg, 0.029 mmol) and 2-amino-2-deoxy-galactose (26 mg, 5 eq.) were dissolved in 2 mL DMF with a drop of TEA. The reaction was stirred at room temperature for 16 h. The polymer was precipitated into diethyl ether from methanol three times. The polymer was dialyzed against water using 1K MWCO tubing to remove excess unreacted amino-sugar and freeze-dried to result in. IR indicated the loss of C=O stretch corresponding to the PFP ester and ^19^F NMR confirmed the loss of the PFP group. This was carried out with 1-amino-1-deoxy-β-d-galactose (Gal1), 2-amino-2-deoxy-β-d-galactose (Gal2), 1-amino-1-deoxy-β-d-mannose (Man1), 2-amino-2-deoxy-β-d-mannose (Man2), 1-amino-1-deoxy-β-d-fucose, as well as ethanolamine as a non-monosaccharide terminated control [26,27]. The isomer1 or isomer2 designation is based on whether the carbohydrate is amino-functionalized at C1 or C2 of glycan ring and attached to the *N*-hydroxyethyl acrylamide polymer by an amide bond (Fig. 1).

### 2.5 Functionalization of 96-well polystyrene plates with monosaccharide-functionalized polymers and fabrication of combinatorial monosaccharide libraries

To make combinatorial libraries of different monosaccharides, a ‘grafting to’ approach was used as we have previously described [[28], [29], [30], [31], [32]] with minor adjustments. The method involves synthesizing a polymer to enable anchoring the monosaccharides of interest to the epoxide-functionalized polystyrene plate. Briefly, the polymer is engineered to have a carbohydrate capture terminus and an immobilization terminus. *N*-Hydroxyethyl acrylamide was polymerized to a degree of polymerization of 20 using reversible addition–fragmentation chain transfer (RAFT) polymerization mediated by a PFP α-terminated RAFT agent (Fig. 1A). Monosaccharides (mannose, galactose, and fucose) were captured onto this polymer scaffold using amino-monosaccharides by simple substitution of the terminal PFP unit. The monosaccharide-functionalized polymers were then immobilized onto 3D-epoxy 96-well polystyrene plates using the ω-terminal thiol from the RAFT agent (Fig. 1B) *via* a simple incubation and washing steps. To achieve this, a 0.1 mg/mL solution of monosaccharide-functionalized pHEA was made up in milliQ water. Solutions of polymers were made up and 100 μL of each of these solutions was applied to a branched and spaced (50 nm thickness) epoxy-functionalized flat-bottom 96-well polystyrene plate and allowed to react for 30 min at room temperature. Each solution was made in triplicate. After 30 min of incubation, the solution was removed, and the well was washed with water three times to remove any unreacted polymer. Various combinations of monosaccharides were prepared, ranging from 100% of one monosaccharide (mannose, galactose, or fucose) to 100% of another (mannose, galactose, or fucose), in steps of 10% to make a total of 11 different wells in triplicate for each library (Supplementary Table 1). This post-polymerization route ensures that all the polymers have the same initial chain length distribution and therefore reduced the variability between the libraries produced but allows for versatile end-group functionalization to make simple libraries with differing carbohydrate densities.

### 2.6 Cell culture and screening of DC response

Each of the monosaccharide-functionalized wells was sterilized with 70% ethanol and excess ethanol was washed with sterile PBS. Immature DCs were incubated in each well for 24 h where each well was seeded with 1 × 10^5^ DCs. Some wells were stimulated with LPS from *E. coli* (10 ng/mL). Control conditions included well with OH-terminated pHEA (i.e. no carbohydrate coating), LPS only stimulation, and unstimulated DCs.

### 2.7 Quantification of indoleamine 2,3-dioxygenase activity

After stimulation, IDO activity was measured by quantification of the levels of kynurenine in the culture supernatant using a colorimetric assay as we have previously described [25,34,35]. The absorbance was determined using a Promega GlowMax Explorer plate reader on a wavelength of 490 nm. The results are plotted against l-kynurenine standards with concentrations ranging from 0 to 200 mM.

### 2.8 Flow cytometry

Cells were harvested from the 96-well plates to FACS tubes and washed with PBA (PBS with 0.5% bovine serum albumin and 0.1% sodium azide) as described previously [13]. DCs were then incubated with labeled antibodies CD274 (APC clone REA1197), CD40 PE (clone HB14), and CD86 FITC (clone FM95) and isotype-matched mouse antibody controls (all from Miltenyi Biotec) for 20 min in the dark at 4 °C. Cells were then washed in PBA twice and fixed with 4% paraformaldehyde until analysis. All samples were analyzed using an FC500 flow cytometer (Beckman Coulter) and data analysis was carried out using Weasel software.

### 2.9 Cytokine quantification

Cell-free culture supernatants were collected and stored at −80 °C before analysis. The levels of cytokines IL-10, IL-12p70, IL-17, IL-4, and IFNγ were measured by sandwich ELISA using the Duo Set ELISA kit (R&D Systems, UK) according to manufacturer's instructions.

### 2.10 Dendritic cells–T cells co-cultures

Autologous co-cultures were carried out with monosaccharides-primed DC as previously described [25,35]. Briefly, T cells were isolated from PBMCs using a pan T cell isolation kit followed by depletion of CD45RO^+^ memory T cells using CD45RO immunomagnetic beads (both from Miltenyi Biotec). Naïve (CD45RO^−^) T cells were co-cultured with DCs at a 1:10 DC to T cell ratio in RPMI1640 supplemented with 5% human AB serum 100 mg/mL streptomycin, 100 U/mL penicillin, and 2 mM l-glutamine (all from Sigma-Aldrich). On day 3, cells were supplemented with IL-2 (2 ng/mL). After six days of co-culture, cells were stimulated with anti-CD3 and anti-CD28 overnight followed by quantifying T cells cytokine profile.

### 2.11 RNA isolation and RT-qPCR

mRNA extraction and isolation were carried out using the RNeasy kit (QIAGEN) and cDNA synthesis was carried out using a qPCR synthesis kit (PCR Biosystems) according to the manufacturer's instructions. Real-time PCR was carried out using SYBR Green qPCR Master Mix (PCRBIOSYSTEMS) and the qPCR cycler Stratagene MxPro 3005P qPCR System (Stratagene, La Jolla, CA). Primers used were GAPDH (forward) 5′-GAGTCAACGGATTTGGTCGT-3′, GAPDH (reverse) 5′-GACAAGCTTCCCGTTCTCAG-3′; FOXP3 (forward) 5′- GTGGCCCGGATGTGAGAAG-3′, FOXP3 (reverse) 5′- GGAGCCCTTGTCGGATGATG-3′; RORγt (forward) 5′- CCTGGGCTCCTCGCCTGACC-3′, RORγt (reverse) 5′- TCTCTCTGCCCTCAGCCTTGCC -3′. The heat cycle was initiated at 95°C for 2 min, followed by 40 cycles of 95°C for 5 s, then 65°C for 25 s and followed by 95°C for 1 min, 55°C 30 sec and 95°C 30 s. Sample values are relative to LPS only and are normalized to the housekeeping gene GAPDH. All samples are run in triplicates.

### 2.12 Statistical analysis

Statistical significance of the data was obtained using GraphPad Prism 7 (GraphPad Software, San Diego, CA). Therefore, paired *t*-tests or ordinary one-way ANOVA was used when comparing between two groups. Statistically significant *p* values were considered to be equal to or lower than 0.05.

## 3 Results

### 3.1 Screening a combinatorial library of immobilized mannose, galactose, and fucose for their ability to modulate DC phenotype

**Fig. 2:**
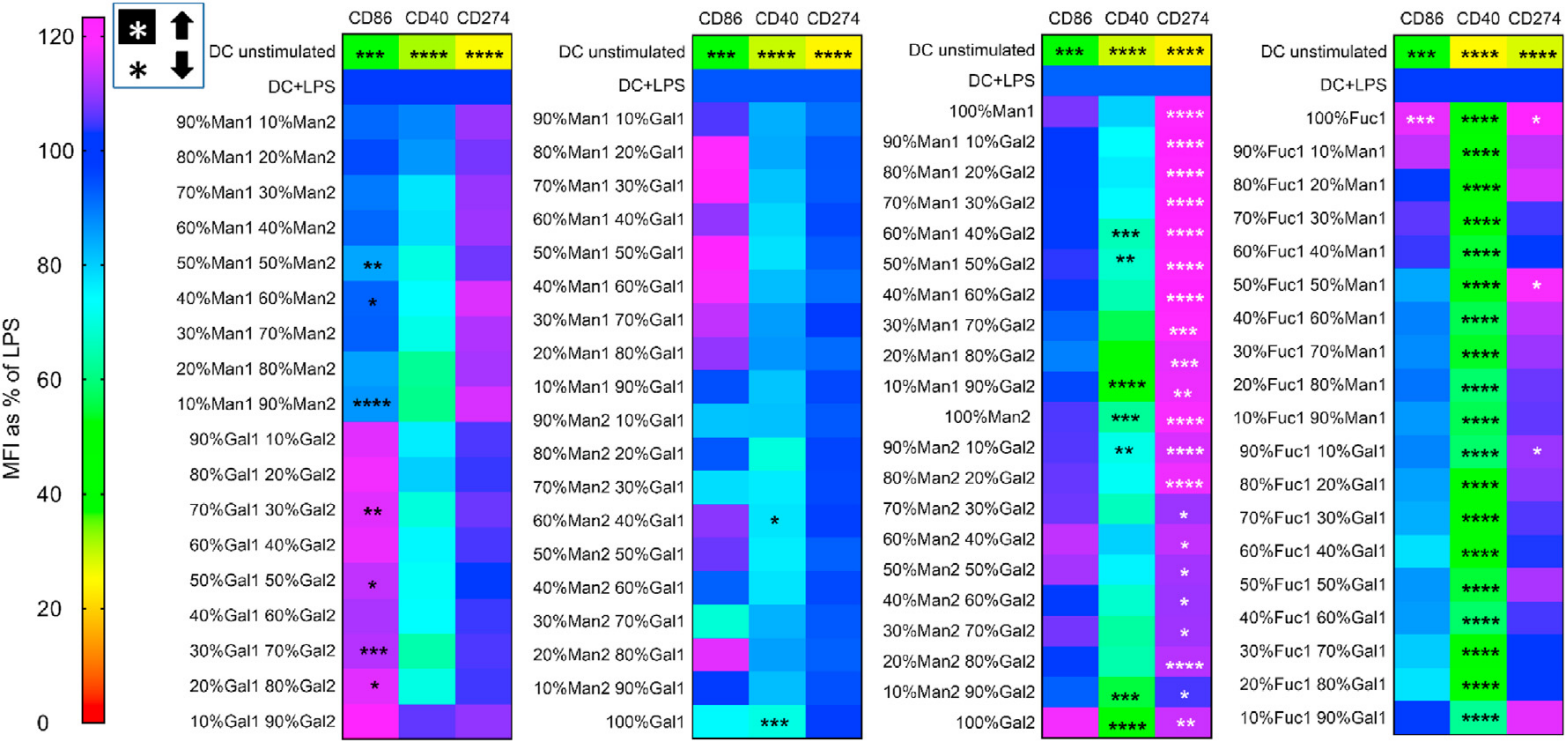
Flow cytometry data are shown as a heat map summarizing the expression profile of three surface markers (CD86, CD40, and CD274) on DCs after incubation on immobilized monosaccharide libraries in the presence of a TLR4 ligand (LPS). Unstimulated (immature) DCs were used as a negative control. All changes are expressed as a percentage of expression levels in mature (LPS-stimulated) DCs. A reduction in CD40 expression was observed in most conditions with the highest reduction in fucose-contained libraries compared to LPS alone. CD86 expression was higher in a number of conditions (particularly mixers of Gal1 and Gal2); however, these did not reach statistical significance. CD274 (PD-L1) expression showed a significant increase in Man1 and Gal2 combinations. Data are shown as mean ± SD of three independent donors where ∗*p*<0.05, ∗∗*p*<0.01, ∗∗∗*p*<0.001, and ∗∗∗∗*p*<0.0001.

**Fig. S3:**
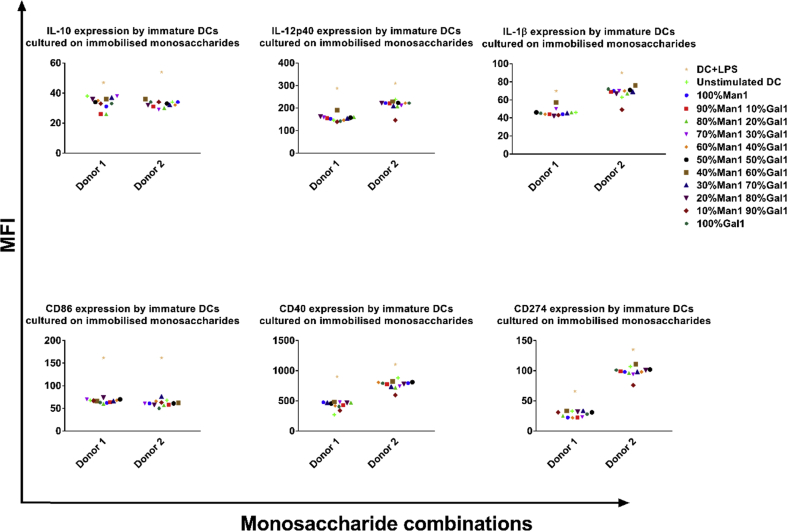
Flowcytometric analysis of DC treated with immobilized monosaccharide libraries without LPS co-stimulation. DCs were analysed for expression of surface markers (CD86, CD40 and CD274), pro-inflammatory cytokines (IL-12p40, IL-1β) and anti-inflammatory cytokine (IL-10). Data show no significant changes induced by monosaccharides in immature DC. Control conditions included un-stimulated DCs and DCs stimulated with LPS only. Data shown are from 2 independent donors.

**Fig. S4:**
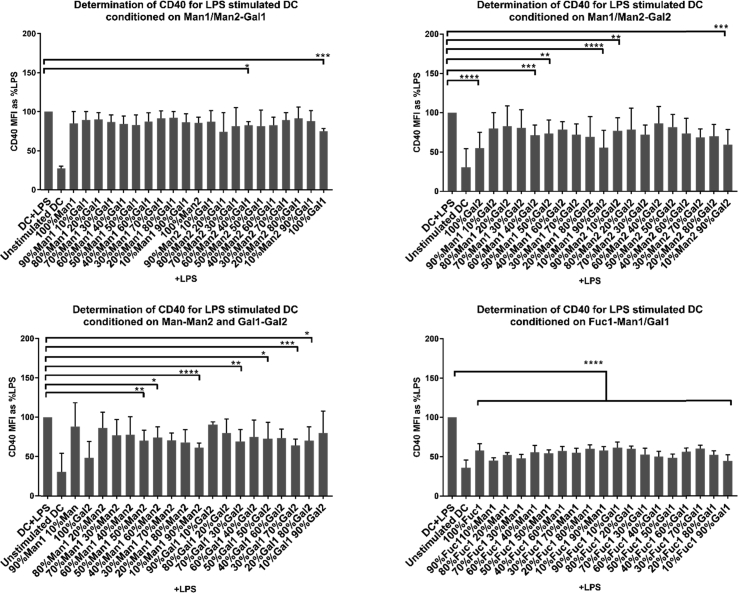
Bar graphs showing expression profile of CD40 on DCs after being cultured on immobilized monosaccharide libraries in the presence of LPS. Unstimulated (immature) DCs were used as a negative control. All changes are expressed as a percentage of expression levels in mature (LPS stimulated) DCs. Data is shown as mean ± SD of three independent donors where *p < 0.05, **p < 0.01, ***p < 0.001, ****p < 0.0001.

**Fig. S5:**
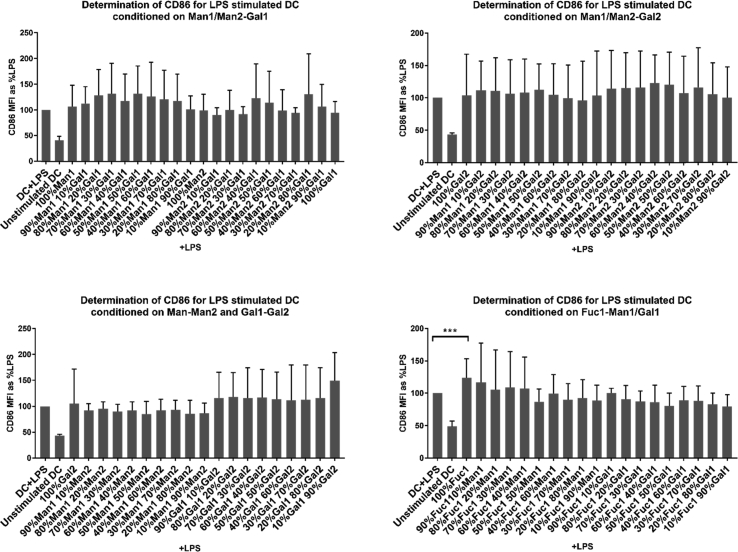
Bar graphs showing expression profile of CD86 on DCs after being cultured on immobilized monosaccharide libraries in the presence of LPS. Unstimulated (immature) DCs were used as a negative control. All changes are expressed as a percentage of expression levels in mature (LPS stimulated) DCs. Data is shown as mean ± SD of three independent donors where *p < 0.05, **p < 0.01, ***p < 0.001, ****p < 0.0001.

**Fig. S6:**
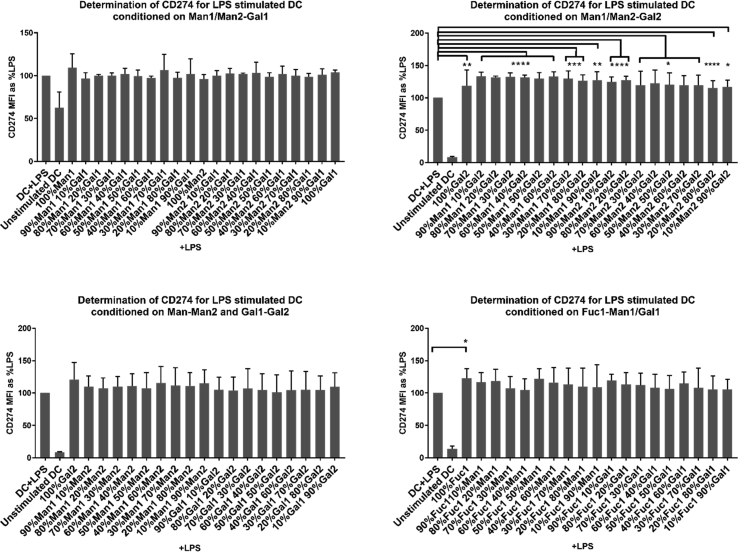
Bar graphs showing expression profile of CD274 on DCs after being cultured on immobilized monosaccharide libraries in the presence of LPS. Unstimulated (immature) DCs were used as a negative control. All changes are expressed as a percentage of expression levels in mature (LPS stimulated) DCs. Data is shown as mean ± SD of three independent donors where *p < 0.05, **p < 0.01, ***p < 0.001, ****p < 0.0001.

**Fig. S7:**
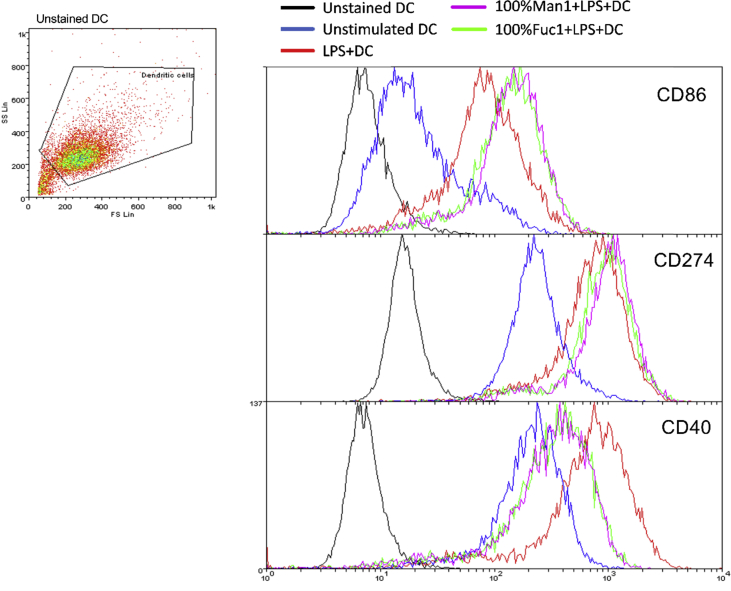
Representative histograms showing expression profile of CD40, CD86 and CD274 for immature and LPS stimulated DCs together with a selection of carbohydrates.

To investigate the effect of surface-immobilized monosaccharides on DC phenotype and IDO activity, immature and LPS-stimulated DCs were cultured on the monosaccharide-immobilized polystyrene plates containing different ratios of amino-fucose, amino-mannose, and amino-galactose (where the number after each glycan indicates the ring-position of the amine, Fig. 1) for 24 h followed by flow cytometric analysis of their surface phenotype and quantifying kynurenine in the supernatant as a surrogate for IDO activity. For analyzing DC phenotype, we chose a selection of surface receptors linked to immune stimulation (CD40 and CD86) or inhibition (CD274 or programmed death ligand 1 (PDL-1)). Unsurprisingly, none of the monosaccharide combinations seemed to influence the immature DC phenotype (Supplementary Fig. 3) since monosaccharides are abundant in the human body contributing to cell membrane physiology and metabolism; hence, without accompanying a potent stimulus (e.g. a danger signal), the immune cells would not react to them [36]. However, flow cytometry data (Fig. 2 and Supplementary Figs. 4–7) showed that 35 immobilized monosaccharides were able to significantly downregulate CD40 expression in LPS-stimulated DCs. Interestingly, all fucose-containing carbohydrates induced significant suppression of CD40 expression. This was in contrast to all Gal1 with Man1 or Man 2 combinations (except 60% Man2 40% Gal1 and 100% Gal1), which did not induce any significant changes in CD40 expression. As indicated previously, the isomer1 or isomer2 designation is based on whether the carbohydrate is functionalized at C1 or C2 of the *N*-hydroxyethyl acrylamide polymer respectively, which seems to have an impact on how they interact with DCs. Furthermore, we observed an increase in CD274 (PD-L1) expression in LPS-stimulated DCs treated with all combinations of Gal2 with Man1 or Man2 indicating a shift toward regulatory/inhibitory DCs. CD86 expression was higher in a number of conditions (particularly combinations of Gal1 and Gal2); however, these did not reach statistical significance (Fig. 2 and Supplementary Figs. 4–6). Nevertheless, at this stage of screening, all monosaccharides, regardless of their impact on DCs’ surface phenotype, were taken forward for additional experimentation.

### 3.2 The effect of immobilized monosaccharides on DC IDO activity

**Fig. 3:**
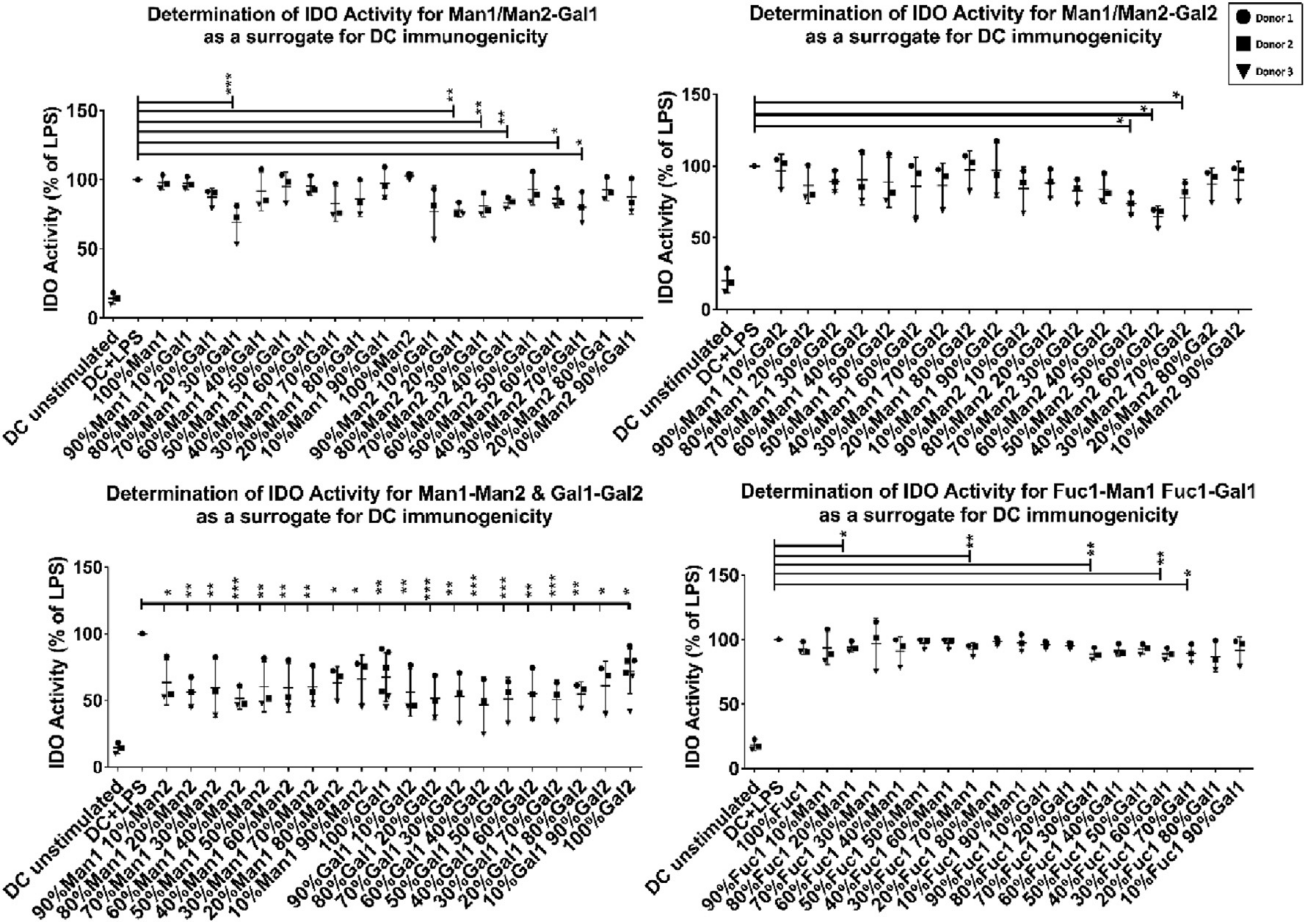
Data obtained from indoleamine 2,3-dioxygenase activity in dendritic cells treated with the immobilized monosaccharides in the presence of a TLR4 ligand (LPS). Reduction in indoleamine 2,3-dioxygenase activity is prominent in monosaccharide combinations consisting of the two isomers of the same monosaccharide while others have a reduction in indoleamine 2,3-dioxygenase activity but not as significant as the latter compared to dendritic cells treated with LPS alone. Data are shown as mean ± SD of three independent donors where ∗*p*<0.05, ∗∗*p*<0.01, ∗∗∗*p*<0.001, and ∗∗∗∗*p*<0.0001.

**Fig. S8:**
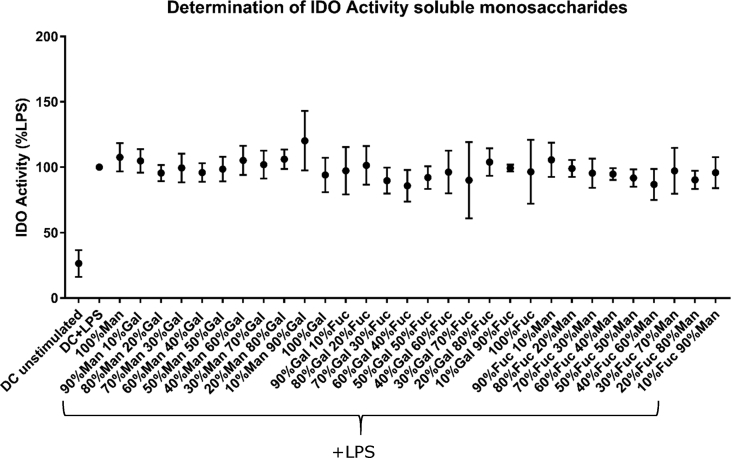
IDO enzymatic activity in response to soluble monosaccharides. DCs were co-stimulated together with 100µg/ml of a mixture of soluble monosaccharides and incubated for 24 hours followed by quantifying IDO activity. No significance change in IDO activity was observed can be seen by the soluble form on DCs enzymatic activity and in turn on the cross talk between TLR4 and CLRs and the activity of the cells compared to LPS alone. Data is shown as mean ± SD of four independent donors.

Next, to understand the impact of monosaccharides on DC immunogenicity, we investigated IDO activity in LPS-stimulated DCs cultured on a selection of monosaccharides predicted to have pro- or anti-inflammatory properties based on their impact on DC phenotype. Data from these experiments show significant reductions in the IDO activity, particularly in combinations mainly constituted of both isoforms of the carbohydrate where the carbohydrate is functionalized at C1 or C2 of the *N*-hydroxyethyl acrylamide polymer for isomer1 or isomer2 respectively (Fig. 3). These findings corroborate observed changes in DCs phenotype (e.g. suppression of CD40 expression) and suggest that DCs primed on a number of immobilized monosaccharides acquire distinct pro- or anti-inflammatory phenotypes [37,38]. Interestingly, similar mixtures of monosaccharides in soluble form did not induce any significant changes in IDO activity (Supplementary Fig. 8).

### 3.3 Immobilized Gal1, Man1–Man2, Man1–Gal2, and Man2–Gal2 significantly increase the production of IL-10 and suppress IL-12 production while Gal1–Gal2 suppresses both cytokines in TLR4-stimulated DCs

**Fig. 4:**
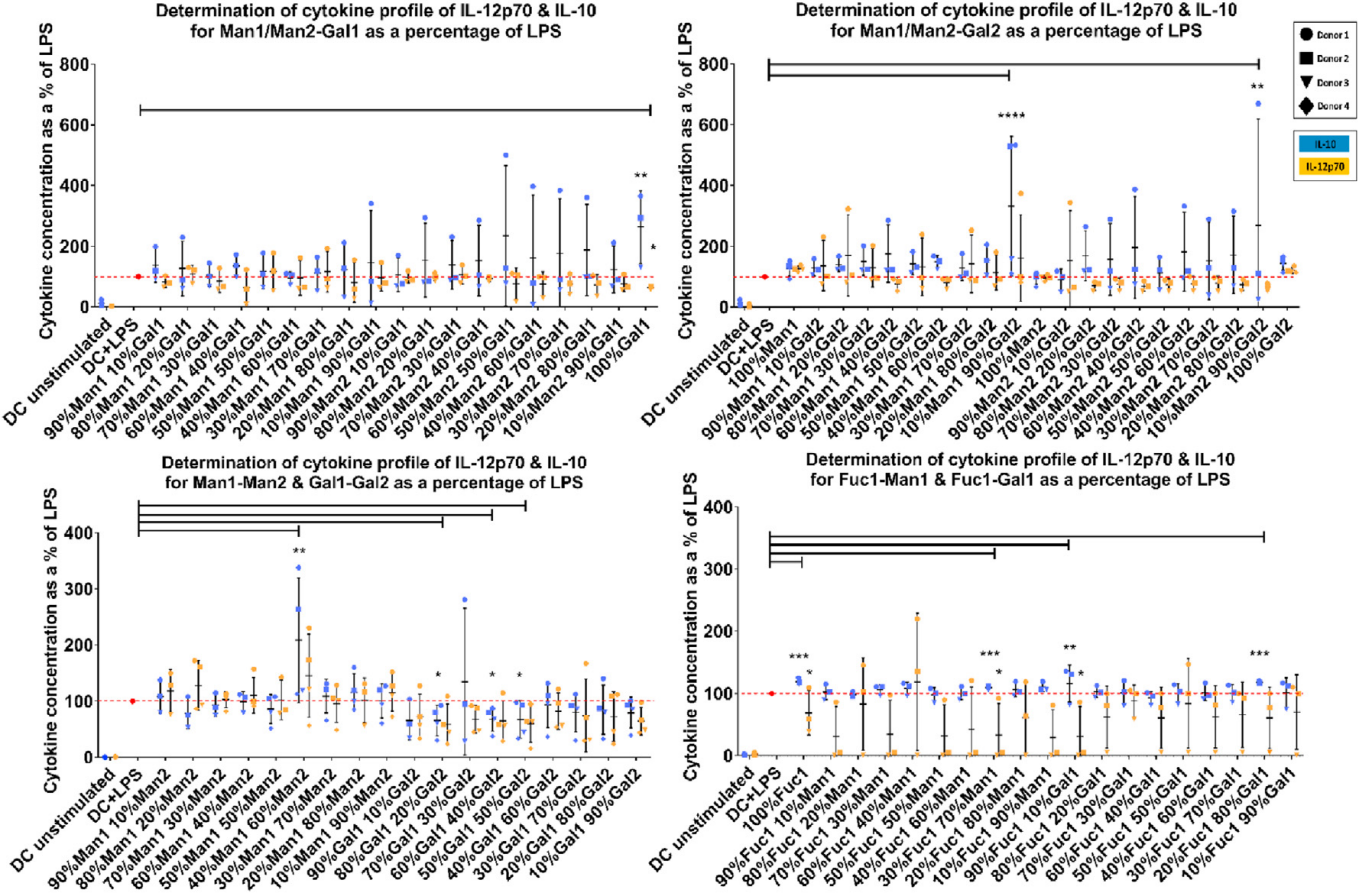
Cytokine profile of dendritic cells treated with immobilized monosaccharides in the presence of a TLR4 ligand (LPS). Gal1, Man1–Man2, Man1–Gal2, and Man2–Gal2 combinations induce a significant increase in IL-10 production and suppression in IL-12 while Gal1–Gal2 suppresses both cytokines in response to LPS stimulation compared to LPS alone. Data are presented as the percent change compared to levels of cytokines produced in response to LPS stimulation for each donor. Data are shown as mean ± SD of ≥3 independent donors where ∗*p*<0.05, ∗∗*p*<0.01, ∗∗∗*p*<0.001, and ∗∗∗∗*p*<0.0001.

**Fig. S9:**
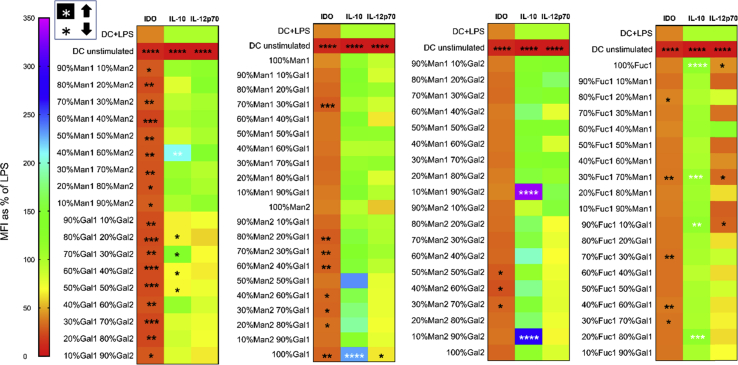
Heatmap graph summarising IDO activity and anti- and pro- inflammatory cytokine profiles of DCs after incubation on immobilized monosaccharide libraries in the presence of LPS. Unstimulated (immature) DCs were used as a negative control. All changes are expressed as a percentage of expression levels in mature LPS stimulated dendritic cells.

To better understand the effect of different monosaccharides shown to modulate DC phenotype and IDO activity on DC function, we also investigated their impact on DC cytokine profile focusing on IL-12 and IL-10 as prototypic pro- and anti-inflammatory cytokines respectively. DCs stimulated with LPS and cultured on 100% galactose1 (Gal1), 40% mannose1 + 60% mannose2 (Man1–Man2), 10% mannose1 + 90% galactose2 (Man1–Gal2), and 10% mannose2 + 90% galactose2 (Man2–Gal2)-coated wells showed a significant increase in IL-10 while suppressing IL-12 production compared to LPS only (Fig. 4 and Supplementary Fig. 9). These findings are in line with the observed changes in the DC phenotype showing suppression in CD40 expression in response to the same monosaccharide combinations. An intriguing finding was identifying sugar combinations (e.g. 50% Gal1 + 50% Gal2 (Gal1–Gal2)) that were able to lower DC IDO activity as well as suppressing both IL-10 and IL-12 levels. These carbohydrate combinations are expected to significantly suppress DC immunogenicity.

### 3.4 Suppressing IDO activity using a competitive inhibitor leads to a similar cytokine profile to monosaccharide-primed DCs

**Fig. 5:**
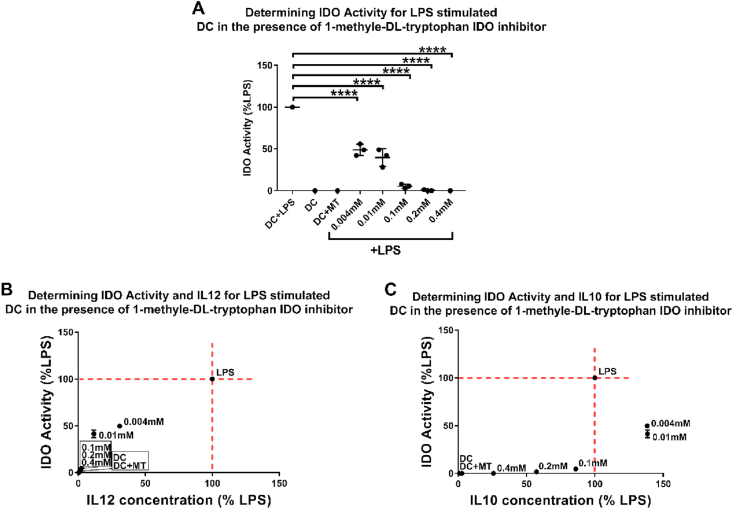
DCs were treated with LPS and varying concentrations of 1-methyl-DL-tryptophan (a competitive indoleamine 2,3-dioxygenase inhibitor) to assess the correlation of IDO activity to IL-10 and IL-12 production. **A**) MT suppresses IDO activity in a dose-dependent manner. **B**) IL-12 production shows a proportional relationship with IDO activity. **C**) IL-10 production shows a peak in lower concentrations of the inhibitor (0.004 mM and 0.01 mM) where IDO is at 50% reduction, while after almost complete inhibition of IDO activity (at 0.4 mM), there is still IL-10, but not IL-12 production. Data presented as mean ± SD of ≥3 independent donors where ∗*p*<0.05, ∗∗*p*<0.01, ∗∗∗*p*<0.001, and ∗∗∗∗*p*<0.0001.

To investigate whether the observed changes in DCs cytokine profile could be due to changes in IDO activity, we treated LPS-stimulated DCs with 1-methyl-DL-tryptophan (MT), a competitive inhibitor of IDO, followed by quantifying IL-12 and IL-10 production [22]. The data show that MT suppressed IDO activity in DCs in a dose-dependent manner (Fig. 5A). The level of reduction in IDO activity in the chosen MT concentration range was comparable with the levels observed in DCs primed with Gal1, Man1–Man2, Man1–Gal2, Man2–Gal2, and Gal1–Gal2. Furthermore, the MT-treated DCs showed a reduction in IL-12 production, similar to their monosaccharide-treated counterparts. These data suggest a correlation between changes in IDO activity and IL-12 production.

Interestingly, in the case of IL-10 in lower concentrations of MT, where IDO activity is only reduced by around 50%, there is an increase in IL-10 production, albeit not statistically significant, which is only suppressed with increasing concentrations of MT and marked reduction in IDO activity to around 10% of LPS only condition. This is consistent with data from monosaccharide-treated DCs showing that while a 50% reduction in IDO activity correlates with a significant suppression of IL-12 production, IL-10 production is increased. Collectively, these data suggest that DC pro- or anti-inflammatory phenotypes correlate with the level of IDO activity where a change in IDO is directly proportional to IL-12 production, whereas IL-10 levels seem to show a biphasic correlation to IDO activity (Fig. 5B and C).

### 3.5 DCs conditioned by Gal1 and Gal1–Gal2 bias T cell differentiation toward IL-10 and IL-17 producing cells respectively

**Fig. 6:**
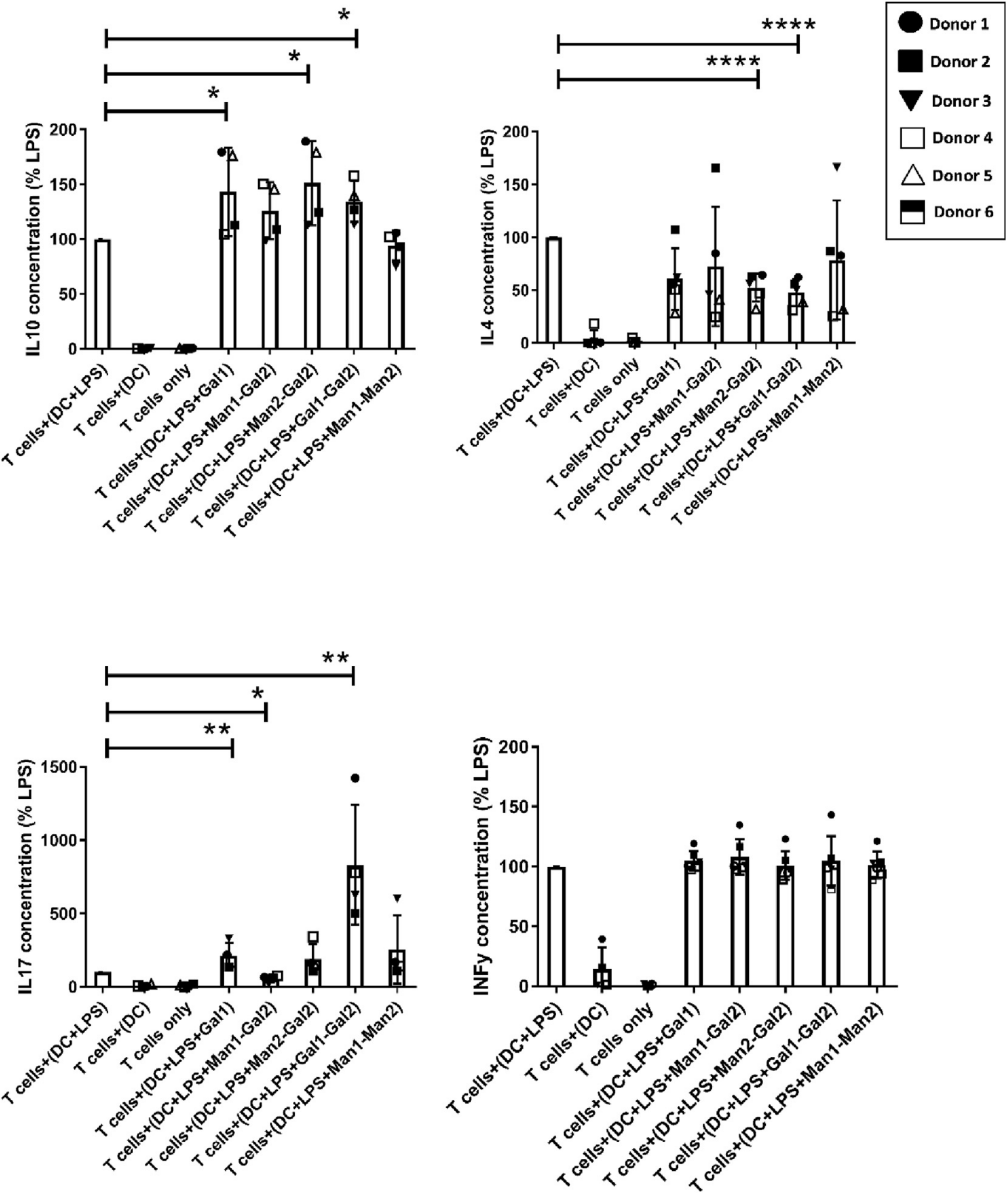
Co-cultures of T cells with monosaccharide-primed DCs were set up to investigate the effect of monosaccharides on T cell differentiation using their cytokine profile. IL-10, IFNγ, IL-4, and IL-17 were selected as prototypic Treg, Th1, Th2, and Th17 cytokines. An increase in IL-10 production can be noted in all conditions reaching statistical significance in Gal1, Gal1–Gal2, and Man2–Gal2. Interestingly, the same conditions also show an increase in IL-17 productions with the highest increase observed in Gal1 and Gal1–Gal2. A significant decrease in IL-17 can be noted for Man1–Gal2 showing a shift of the immune response away from Th17 and into Th1. Data are shown as mean ± SD of ≥4 independent donors where ∗*p*<0.05, ∗∗p<0.01, ∗∗∗*p*<0.001, and ∗∗∗∗*p*<0.0001.

Gal1, Man1–Man2, Man1–Gal2, Man2–Gal2, and Gal1–Gal2 modulated DC surface stimulatory ligands, cytokine production, and IDO activity in LPS-stimulated DCs suggesting a regulatory phenotype. These conditions were selected for investigating downstream effects on T cell differentiation in DC–T cell co-culture studies. Co-cultures were carried out using autologous naïve T cells and DCs, where DCs were primed with the selected immobilized carbohydrates in the presence of LPS for 24 h and then co-cultured with naïve T cells for six days. Data show a significant increase in IL-10 production by naïve T cells in Gal1, Gal1–Gal2, and Man2–Gal2 conditions (Fig. 6). An interesting finding was that T cells co-cultured with Gal1 and Gal1–Gal2 treated DCs showed a significant increase in IL-17 production compared to LPS alone, whereas a decrease in IL-17 can be noted in Man1–Gal2. These data also show a significant decrease in IL-4 production by T cells co-cultured with Gal1–Gal2 and Man2–Gal2 treated DCs while IFN-γ levels remain unchanged compared to LPS alone for all conditions.

**Fig. 7:**
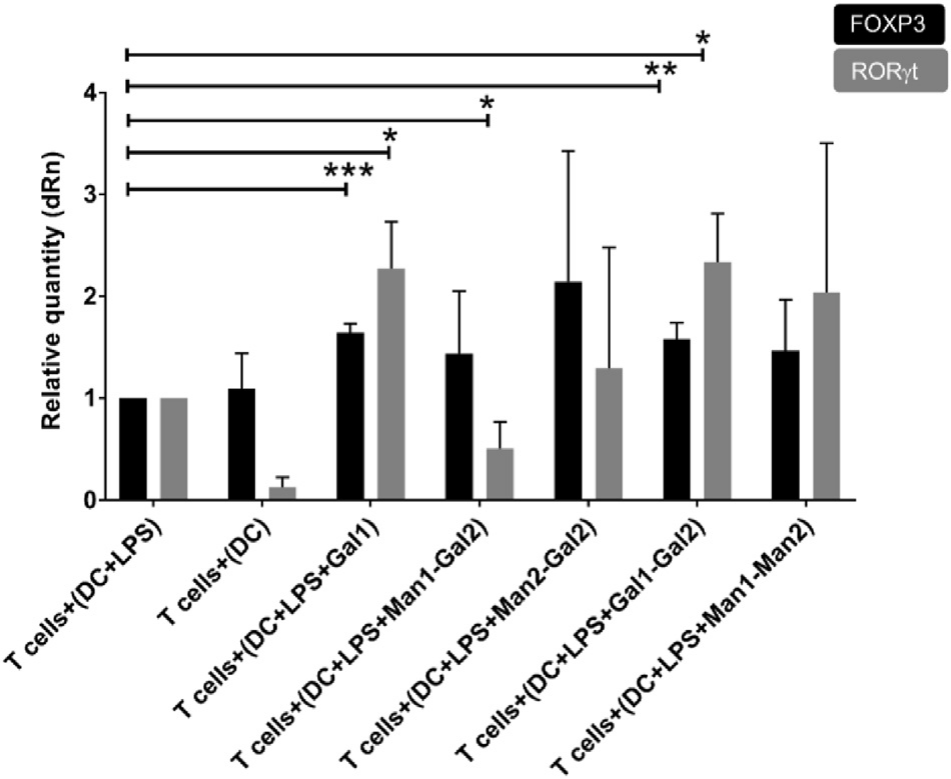
RT-PCR experiments to quantify FOXP3 and RORγt expression to further confirm the T cell phenotype. A significant increase in FOXP3 expression can be seen in Gal1 and Gal1–Gal2. Interestingly, a significant increase in RORγt production can be noted in Gal1–Gal2 and Gal1, whereas a reduction is observed in Man1–Gal2 indicating a possible presence of Th17 and Tregs in the population for Gal1–Gal2 and Gal1 and the absence of Th17 in Man1–Gal2 condition. Data are shown as mean ± SD of triplicates of three independent donors.

From these data, we hypothesized that naïve T cells co-cultured with Gal1 and Gal1–Gal2 treated DCs have acquired mixed anti-inflammatory and pro-inflammatory phenotypes similar to what has been recently described in the context of some inflammatory conditions and cancers [39]. To further confirm the phenotype of these cells, we investigated the expression of FOXp3 and RORγt, major Treg, and Th17 transcription factors respectively, using real-time polymerase chain reaction (RT-PCR). We were able to confirm the expression of both FOXp3 and RORγt, indicating the generation of cells with a mixed Treg/Th17 phenotype on immobilized Gal1 and Gal1–Gal2 (Fig. 7). Interestingly, Man1–Gal2 showed a decrease in IL-17 production and RORγt gene expression, indicating that the inclusion of mannose changed cell differentiation away from Th17 and toward Th1 as evidenced by a significant decrease in IL-17 and unchanged levels of IFN-γ.

## 4 Discussion

There is increasing interest in biomaterials that are able to modulate immune responses to promote healing and suppress adverse immune responses with a significant focus on modulating macrophage phenotype [40]. There are encouraging data from the use of polymers, small molecules, and materials with defined shape and topographies that are able to dampen adverse immune responses [[41], [42], [43], [44]]. However, despite its importance in determining the fate of implanted materials [45], controlling adaptive immune responses through material engineering has remained relatively unexplored. The potent immune-modulatory properties of carbohydrates and many tools available for their modification and incorporation into existing materials make them attractive candidates for engineering new materials with immune-instructive properties.

The central role of DCs in shaping adaptive immune responses means that these cells are ideal targets for immune modulation including controlling immune responses against implanted materials [46]. Carbohydrates decorating pathogens and tumor cells have been known to trigger protective or inhibitory immune responses by modulating DC function, which typically underpin immune evasion or survival of tumors [47,48]. The latter is mainly through skewing DCs’ response from inflammatory to tolerogenic, which in turn suppresses the immune responses against tumor cells [49]. This is in line with our data showing that a number of ‘hit’ conditions (e.g. Gal1, Gal1–Gal2, Man1–Gal2, Man2–Gal2, and Man1–Man2) were able to induce a regulatory phenotype in DCs as evidenced by a significant reduction in the expression of co-stimulatory molecule CD40 and increase in tolerogenic CD274 (PDL-1) expressions in LPS-challenged DCs. CD40 has been shown to play an important role in supporting pro-inflammatory responses and Th1 differentiation through engaging CD154 (or CD40L) on T cells which is known to induce high levels of IL-12 production, a key pro-inflammatory cytokine [38,50], whereas CD274 (PDL-1) is an inhibitory ligand for T cell activation with a role in Treg development [51]. This is also consistent with how some pathogens modulate the immune system in their favor. For example, Hamilton et al. showed that *Fasciola hepatica* tegumental glyco-antigens suppress DC maturation by decreasing CD40 expression in DCs, contributing to the pathogen survival [52]. Interestingly, our data also showed that the same group of monosaccharides that suppressed CD40 expression and induced CD274 (PDL-1) expression could significantly suppress LPS-induced IDO activity in DCs. IDO is the rate-limiting enzyme for the catabolism of essential amino acid tryptophan and is known for its modulatory effects on immune cells and importantly on DCs through different mechanisms such as depletion of tryptophan and producing immune-regulatory metabolites like kynurenine [13,35]. Despite some contradictory evidence on the correlation between IDO activity and DCs function, it is clear that suppression of IDO activity and reduced tryptophan levels in the microenvironment suppress inflammatory immune responses and could promote immune regulation [[53], [54], [55], [56]]. This is also in line with studies showing increased levels of IDO activity in tumor environments, which leads to depletion of tryptophan causing DCs to switch to anergic state and, ultimately, survival of the tumor cells [57,58]. To understand the importance of monosaccharides immobilization in the observed effects on DCs, we also treated DCs with a selection of monosaccharide combinations in soluble form. These experiments showed that none of the soluble monosaccharide combinations had a significant impact on IDO activity (Supplementary Fig. 8). These observations suggest that receptor clustering or conformation of monosaccharides play an important role in their immune-modulatory effects on DCs. Nevertheless, in a large group of pathologies, including autoimmune and inflammatory diseases as well as adverse pro-inflammatory responses to implanted materials, ‘regulatory’ monosaccharides that are able to suppress IDO activity and promote tolerogenic DCs to suppress excessive inflammation will be highly desirable and could play a significant role in developing new therapeutic strategies for these groups of pathologies [46,59,60].

Changes in DCs phenotype and IDO activity in ‘hit’ monosaccharide-primed DCs are also reflected in their pro- and anti-inflammatory cytokine profile. Specifically, LPS-stimulated DCs cultured on Gal1, Gal1–Gal2, Man1–Gal2, Man2–Gal2, and Man1–Man2 showed a significant suppression in IL-12 production and an increase in IL-10. This is in line with how glycoantigens in some pathogens modulate DC function. For example, ManLam is a component of *Mycobacterium tuberculosis* cell membrane that is terminated in mannose and its interaction with DC-SIGN has been shown to suppress DC function through an increase in IL-10 and a lower IL-12 production [61]. IL-12 is a potent pro-inflammatory cytokine that plays a key role in immune response against intracellular pathogens and Th1 cell differentiation, whereas IL-10 is a signature anti-inflammatory cytokine that promotes Treg differentiation and is also produced by these cells playing a key role in maintaining immune homeostasis and resolution of inflammation after injury [62]. These data therefore strongly suggest that the ‘hit’ monosaccharide-primed DCs have acquired a tolerogenic phenotype, which was further confirmed in DC–T cell co-cultures where conditioned DCs were shown to support naïve T cell differentiation toward Treg phenotype. It should be noted that the relatively high level of inter-individual variation in cytokine levels between different donors meant that despite some marked changes in IL-12 or IL-10 levels in some donors, these changes did not reach statistical significance. This could be due to the presence of both high and low responders to monosaccharide combinations and/or LPS within the donor population [63]. To account for inter-individual variation between different donors, we normalized data for each donor to their data from LPS stimulation.

The ability of ‘hit’ monosaccharide-primed DCs in instructing downstream adaptive responses highlights the biological relevance of the monosaccharide-conditioned DCs. Using engineered materials to modulate DC function has gained traction in the last few years with promising results in areas like DC-based cancer vaccination [64,65]. The ability to generate tolerogenic DCs and skew T cell responses toward Treg using immobilized monosaccharide combinations such as Gal1, Gal1–Gal2, Man1–Gal2, Man2–Gal2, and Man1–Man2, therefore, provides exciting opportunities for using materials ‘decorated’ by a selection of monosaccharides for promoting immune regulation in the context of inflammatory and autoimmune diseases as well as implanted materials. Conditions that showed a reduction in CD40 and IDO activity together with increased IL10 or a decrease in both IL12 and IL10 were selected as tolerogenic DCs and were taken forward for co-culture with DCs.

T cells’ plasticity and their ability to respond to different signals and converging distinct signaling pathways are a widely accepted concept and provide opportunities for fine-tuning their phenotype [66]. Interestingly, the Gal1–Gal2 combination supported a significant increase in both IL-17 and IL-10 production. This is in line with previous work that has described regulatory T cells with the capacity to produce IL-17 to be highly immune suppressive and are believed to be related to tumor survival [67]. While future work should focus on better understanding of the impact of monosaccharides on T cell polarization and contribution of DCs in the observed increase in IL-10 production, the present data suggest that the Gal1–Gal2 combination could prove a useful tool for developing new immunotherapies against inflammatory conditions where aberrant immune activation underpins tissue damage. Intriguingly, inclusion of mannose to either of Gal isoforms led to the suppression of IL-17 production while maintaining high IL-10 levels. This is broadly in line with the other studies showing that mannose moieties support IL-10 production [61,68].

## 5 Conclusions

In this study, we screened a combinatorial library of immobilized monosaccharides for their ability to modulate phenotypical and functional properties of DCs. This resulted in identifying a number of amino-monosaccharides (galactose and mannose with amines at positions 1 or 2; Gal1, Man1–Gal1, Man1–Gal2, and Gal1–Gal2) that induce differentiation of tolerogenic DCs, able to support naïve T cell differentiation toward Treg phenotype. Our data also show that 1-amino galactose and 2-amino galactose (Gal1–Gal2)-primed DCs support Tregs that also produce high levels of IL-17, a T cell phenotype that is expected to be highly immune suppressive. These data will provide a basis for the rational design of monosaccharide-coated surfaces with immune-regulatory properties with the ability to fine-tune innate and adaptive immune responses with applications in materials-based immunotherapy for inflammatory conditions and implanted medical devices where suppressing adverse immune responses is desirable. Future work should focus on investigating the efficacy of ‘hit’ monosaccharides in preclinical models.

## Data availability

All relevant data are available from the University of Nottingham's Research Data Management Repository https://rdmc.nottingham.ac.uk.

## Credit author statement

MAA carried out experimental work, analyzed data, and wrote the original draft of the manuscript. SJR carried out the synthesis and fabrication of the immobilized monosaccharide libraries. AMG conceptualized the study. MRA, MIG, and AMG supervised the study, wrote and revised the manuscript. All authors approved the final version of the manuscript.

## Declaration of competing interest

The authors declare that they have no known competing financial interests or personal relationships that could have appeared to influence the work reported in this article.

## Appendix A Supplementary data

Multimedia component 1 List of monosaccharide combination library used for screening. Carbohydrate1 and carbohydrate2 are isoforms and are attached to either C1 or C2 of the polymer respectively. Each column consists of carbohydrate combinations of 10% imcrements in one carbohydrate and decrements in the other carbohydrate creating a unique combination of monosaccharide conjugated polymers. Multimedia component 1
