# Supplementary material for: Developing immune-regulatory materials using immobilized monosaccharides with immune-instructive properties
Source: Mater Today Bio. 2020 Sep 30;8:100080. doi: 10.1016/j.mtbio.2020.100080 (PMC7649522; doi:10.1016/j.mtbio.2020.100080)
Supplement: Multimedia component 1 — List of monosaccharide combination library used for screening. Carbohydrate1 and carbohydrate2 are isoforms and are attached to either C1 or C2 of the polymer respectively. Each column consists of carbohydrate combinations of 10% imcrements in one carbohydrate and decrements in the other carbohydrate creating a unique combination of monosaccharide conjugated polymers. [file mmc1.docx]

# Supplementary data

| Monosaccharide combinations | | | |
| --- | --- | --- | --- |
| 100%Mannose1 | 100%Mannose1 | 100%Galactose1 | 100%Fucose1 |
| 90% Mannose1 10%Galactose1 | 90% Mannose1 10% Galactose2 | 90% Galactose1 10% Galactose2 | 90% Fucose1 10%Mannose1 |
| 80% Mannose1 20% Galactose1 | 80% Mannose1 20% Galactose2 | 80% Galactose1 20% Galactose2 | 80% Fucose1 20% Mannose1 |
| 70% Mannose1 30% Galactose1 | 70% Mannose1 30% Galactose2 | 70% Galactose1 30% Galactose2 | 70% Fucose1 30% Mannose1 |
| 60% Mannose1 40% Galactose1 | 60% Mannose1 40% Galactose2 | 60% Galactose1 40% Galactose2 | 60% Fucose1 40% Mannose1 |
| 50% Mannose1 50% Galactose1 | 50% Mannose1 50% Galactose2 | 50% Galactose1 50% Galactose2 | 50% Fucose1 50% Mannose1 |
| 40% Mannose1 60% Galactose1 | 40% Mannose1 60% Galactose2 | 40% Galactose1 60% Galactose2 | 40% Fucose1 60% Mannose1 |
| 30% Mannose1 70% Galactose1 | 30% Mannose1 70% Galactose2 | 30% Galactose1 70% Galactose2 | 30% Fucose1 70% Mannose1 |
| 20% Mannose1 80% Galactose1 | 20% Mannose1 80% Galactose2 | 20% Galactose1 80% Galactose2 | 20% Fucose1 80% Mannose1 |
| 10% Mannose1 90% Galactose1 | 10% Mannose1 90% Galactose2 | 10% Galactose1 90% Galactose2 | 10% Fucose1 90% Mannose1 |
| 100% Galactose1 | 100% Galactose2 | 100% Galactose2 | 100% Mannose1 |
| 100%Mannose2 | 100%Mannose2 | 100%Mannose1 | 100%Fucose1 |
| 90% Mannose2 10%Galactose1 | 90% Mannose2 10% Galactose2 | 90% Mannose1 10% Mannose2 | 90% Fucose1 10%Galactose1 |
| 80% Mannose2 20% Galactose1 | 80% Mannose2 20% Galactose2 | 80% Mannose1 20% Mannose2 | 80% Fucose1 20% Galactose1 |
| 70% Mannose2 30% Galactose1 | 70% Mannose2 30% Galactose2 | 70% Mannose1 30% Mannose2 | 70% Fucose1 30% Galactose1 |
| 60% Mannose2 40% Galactose1 | 60% Mannose2 40% Galactose2 | 60% Mannose1 40% Mannose2 | 60% Fucose1 40% Galactose1 |
| 50% Mannose2 50% Galactose1 | 50% Mannose2 50% Galactose2 | 50% Mannose1 50% Mannose2 | 50% Fucose1 50% Galactose1 |
| 40% Mannose2 60% Galactose1 | 40% Mannose2 60% Galactose2 | 40% Mannose1 60% Mannose2 | 40% Fucose1 60% Galactose1 |
| 30% Mannose2 70% Galactose1 | 30% Mannose2 70% Galactose2 | 30% Mannose1 70% Mannose2 | 30% Fucose1 70% Galactose1 |
| 20% Mannose2 80% Galactose1 | 20% Mannose2 80% Galactose2 | 20% Mannose1 80% Mannose2 | 20% Fucose1 80% Galactose1 |
| 10% Mannose2 90% Galactose1 | 10% Mannose2 90% Galactose2 | 10% Mannose1 90% Mannose2 | 10% Fucose1 90% Galactose1 |
| 100% Galactose1 | 100% Galactose2 | 100% Mannose2 | 100% Galactose1 |

**Supplementary data Table1:**

List of monosaccharide combination library used for screening. Carbohydrate1 and carbohydrate2 are isoforms and are attached to either C1 or C2 of the polymer respectively. Each column consists of carbohydrate combinations of 10%imcrements in one carbohydrate and decrements in the other carbohydrate creating a unique combination of monosaccharide conjugated polymers.
